# Acute Alterations of Somatodendritic Action Potential Dynamics in Hippocampal CA1 Pyramidal Cells after Kainate-Induced Status Epilepticus in Mice

**DOI:** 10.1371/journal.pone.0026664

**Published:** 2011-10-24

**Authors:** Daniel Minge, Robert Bähring

**Affiliations:** Institut für Zelluläre und Integrative Physiologie, Zentrum für Experimentelle Medizin, Universitätsklinikum Hamburg-Eppendorf, Hamburg, Germany; Georgia State University, United States of America

## Abstract

Pathophysiological remodeling processes at an early stage of an acquired epilepsy are critical but not well understood. Therefore, we examined acute changes in action potential (AP) dynamics immediately following status epilepticus (SE) in mice. SE was induced by intraperitoneal (i.p.) injection of kainate, and behavioral manifestation of SE was monitored for 3–4 h. After this time interval CA1 pyramidal cells were studied *ex vivo* with whole-cell current-clamp and Ca^2+^ imaging techniques in a hippocampal slice preparation. Following acute SE both resting potential and firing threshold were modestly depolarized (2–5 mV). No changes were seen in input resistance or membrane time constant, but AP latency was prolonged and AP upstroke velocity reduced following acute SE. All cells showed an increase in AP halfwidth and regular (rather than burst) firing, and in a fraction of cells the notch, typically preceding spike afterdepolarization (ADP), was absent following acute SE. Notably, the typical attenuation of backpropagating action potential (b-AP)-induced Ca^2+^ signals along the apical dendrite was strengthened following acute SE. The effects of acute SE on the retrograde spread of excitation were mimicked by applying the Kv4 current potentiating drug NS5806. Our data unveil a reduced somatodendritic excitability in hippocampal CA1 pyramidal cells immediately after acute SE with a possible involvement of both Na^+^ and K^+^ current components.

## Introduction

Action potential (AP) firing and spread of excitation are highly diverse in the central nervous system, since AP amplitude, waveform and mode of propagation can be regulated by a variety of ion channel conductances [1], [2]. Moreover, neuronal excitability and discharge behavior may change during development [3] and in an activity-dependent manner [4] due to intrinsic and synaptic plasticity processes.

In individual neurons transient and lasting Nav current components may overlap and act in concert with different types of voltage-dependent Ca^2+^ (Cav) and K^+^ (Kv) currents, with currents mediated by Ca^2+^-dependent K^+^ channels of big (BK, activated by both voltage and cytoplasmic Ca^2+^) and small conductance (SK, activated only by cytoplasmic Ca^2+^) and with a hyperpolarization-activated cationic current (*I*_h_) to shape the AP waveform [1]. Ionic conductances may influence AP firing by acting in the subthreshold and/or suprathreshold range of membrane potentials. In particular, during moderate membrane depolarization a delayed and dendrotoxin-sensitive K^+^ current (*I*_D_; [5], [6]) and a subthreshold-activating somatodendritic A-type K^+^ current (*I*_SA_; [7], [8]) may reduce the chance of AP initiation. On the other hand, low-voltage-activated (LVA) Ca^2+^ currents [9] and *I*_h_ [10] may favor AP initiation, thereby inducing oscillatory and pacemaker discharge behavior. Once an AP has been fired, various Kv currents, including *I*_D_ and *I*_SA_ [5], [11], mediate repolarization. This process may be delayed whenever high-voltage-activated (HVA) Ca^2+^ currents are activated by the AP, however, the Ca^2+^ influx may also cause fairly rapid activation of BK currents, and eventually favor repolarization [12]. More slowly reacting to a rise in internal Ca^2+^, SK currents may cause afterhyperpolarization (AHP; [13]) together with the BK current [14] and a muscarinic receptor-inhibited Kv current (M-current; [15]). Finally, not only AHP but also afterdepolarization (ADP) can be frequently observed. ADP may be mediated by a combination of “persistent” (non-inactivating) and “resurgent” (transiently active during recovery from inactivation) Nav current components [16], but also by LVA and R-type (dihydropyridine-resistant) Cav currents [17], [18] and Ca^2+^-activated cationic currents [19].

APs generated at the soma travel down the axon in order to depolarize presynaptic terminals, but they may also be actively propagated, in a retrograde manner, along the apical dendrite [20], [21]. AP backpropagation is often counteracted and finally terminated by dendritic K^+^ efflux. In this context *I*_SA_ plays a central role. The subthreshold activation of *I*_SA_ and the fact that *I*_SA_ increases with distance from the soma [22] leads to progressive attenuation of backpropagating APs (b-APs) in CA1 pyramidal cells [22].

Lasting changes in cellular excitability, discharge behavior and/or spread of excitation (i.e. intrinsic plasticity) are observed in many CNS disorders, including epilepsy [4]. They may be caused by the pathophysiological remodeling of one or more of the ion channel conductances described above. Molecular remodeling processes in individual neurons become more and more important concerning the question: how does a normal brain develop epilepsy after an insult or injury [23]? To study the molecular remodeling processes during the development of chronic epilepsy rodent models have been in use for a long time [24]–[26]. In many of these models status epilepticus (SE) is induced by application of convulsant drugs like kainate [24] or pilocarpine [25]. SE is self-terminating due to postictal depression or terminated by the use of sedative drugs, usually within a few hours. The brief SE period is followed by a longer time interval (weeks or months) with no signs of epilepsy (latent period) before spontaneous recurrent seizures can be observed. After the latent period burst firing [27] and reduced b-AP attenuation [28], as two major correlates of intrinsic plasticity in CA1 pyramidal cells of the hippocampus, have been described.

Investigations with the objective of identifying molecular remodeling processes which underlie epilepsy are usually undertaken at a time point, when considerable cell death has already occurred [29]. This may preclude the unambiguous characterization of intrinsic plasticity mechanisms. To make predictions of the onset and time course of epileptogenesis one has to know the cellular plasticity processes involved at an early stage of the disease. Therefore, we studied possible changes in excitability, AP waveform and b-AP attenuation as correlates of intrinsic plasticity in hippocampal CA1 pyramidal cells immediately after a 1–2 h kainate-induced SE (acute SE) in mice. Our experimental results suggest an acute SE-related modulation of AP dynamics, including reduced somatodendritic excitability and less spread of dendritic excitation.

## Methods

### Animals and status epilepticus induction

**Figure 1:**
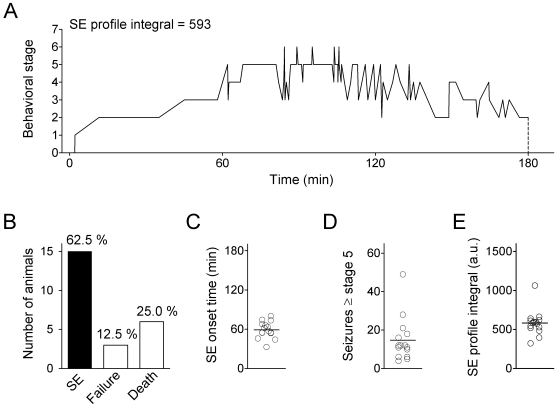
Monitoring status epilepticus induction in mice. Status epilepticus (SE) was induced by an intraperitoneal (i.p.) injection of kainate in juvenile (3 weeks old) C57BL/6 mice. **A**. Typical SE profile (behavioral stage transitions over time); kainate was injected at time point 0, and the animal was sacrificed after 180 min). Behavioral analysis was based on an 8 point scale (0 to 7; see Methods). SE severity was quantified by the SE profile integral (area under SE profile curve between 0 and 180 min, number in arbitrary units; see Methods). **B**. Rate of successful SE induction (n = 15), failure (n = 3) and death (n = 6) within the group of all kainate-injected animals (n = 24). **C**. SE onset time (time until stage 5 was first reached, n = 15). **D**. Number of seizures ≥ stage 5 (n = 15). **E**. SE profile integral (a.u., arbitrary units; n = 15). Mean values are indicated by horizontal lines.

For the experiments juvenile (3 weeks old) C57BL/6 mice were used with a mean body weight of 10.8±0.3 g (n = 24). Status epilepticus (SE) was induced by intraperitoneal (i.p.) injection of kainate (30 mg/kg body weight) diluted in a 0.9% NaCl solution (injection volume: 0.05 ml/g body weight). Seizure onset, duration and severity were monitored by video-taping for 3 h (in one animal for 4 h). Behavioral observations during this time were scored on a modified Racine-scale from 0 to 7 [30], [31]. According to this scale, stage 0 represents normal behavior (exploring, sniffing and grooming). Stage 1 (motionless behavior, cower, flattened ears and facial clonus) was usually followed by stage 2, in which the animals show stretched tail and forelimbs. At stage 3 the animals show various automatisms including repetitive scratching, cycling and head nodding, combined with occasional forelimb cloni, and stage 4 is defined by rearing with continuous forelimb cloni. Finally, stage 5 is defined as rearing and falling, and stage 6 by full motor seizures, sometimes with sudden jumps (stage 6 phases usually lasted less than 30 s in our experiments; full motor seizures longer than 30 s usually caused the death of the animal, defined as stage 7). All stage transitions due to sudden or gradual changes in behavior during the 3–4 h post-injection period were plotted against time and the corresponding data points connected by a line (SE-profile; Fig. 1A). The area under the resultant curve (SE profile integral in arbitrary units) and the number of seizures ≥ stage 5 served as a measure of SE severity to compare all tested animals of the basic population. Animals which received an i.p. injection of 0.9% NaCl solution without kainate (*sham* treatment, n = 10; mean body weight 10.4±0.5 g) yielded reference data (see below) and were not behaviorally analyzed.

### Ethics statement

All animal experiments were approved by the local authorities of the City of Hamburg (approval numbers G09/085, ORG468) and were conducted in accordance with the German law on the protection of experimental animals and the European Communities Council Directive of November 24 1986 (86/609/EEC).

### Hippocampal slice preparation

Acute hippocampal slices were prepared 3–4 h after kainate injection, provided the animal showed clear SE behavior (see above), or after 3 h of *sham* treatment. Animals were anesthetized with isoflurane and sacrificed by decapitation. Brains were quickly removed and transferred into ice-cold sucrose-based cutting solution, containing (in mM) 87 NaCl, 25 NaHCO_3_, 1.25 NaH_2_PO_4_, 2.5 KCl, 0.5 CaCl_2_, 4 MgCl_2_, 25 glucose, 75 sucrose, for 1–2 min. After removal of the cerebellum the two hemispheres were separated, and each hemisphere trimmed and mounted on a VT1000 vibratome (Leica) according to the “magic cut” procedure [32]. With this procedure the blade of the vibratome hits the medial part of the hippocampal formation in a 90° angle and, thus, runs parallel to the CA1 pyramidal cell apical dendrites in this region. Both hemispheres were cut into slices of 300 µm thickness, and the hippocampal formation was separated from the rest of the slice. Hippocampal slices were transferred to a chamber with cutting solution at 34°C for 30 min and stored thereafter in cutting soultion at room temperature (20–22°C). For the experiments slices were transferred to a bath solution at room temperature containing (in mM) 125 NaCl, 26 NaHCO_3_, 1.25 NaH_2_PO_4_, 2.5 KCl, 2.5 CaCl_2_, 1.5 MgCl_2_, 25 glucose. Cutting and bath solutions were kept saturated with carbogen gas (95% O_2_ and 5% CO_2_) at all times.

### Dye filling of neurons and current-clamp recording

Individual CA1 pyramidal cells were visualized with an Axioskop 2F microscope (Zeiss) equipped with infrared differential interference contrast and an iXon CCD Camera (Andor Technology). Patch pipettes were made from thick-walled borosilicate glass (GB150-8P, Science Products). Before pipette filling the fluorescent dye bis-Fura 2 (0.1 mM) was added to the pipette solution, which contained (in mM) 120 K-methylsulfonic acid, 20 KCl, 2 MgCl_2_, 10 HEPES, 4 Na_2_-ATP, 0.3 Na-GTP, 10 Na_2_-phosphocreatine, 3 Na-L-ascorbate; pH 7.2 with KOH. Cells were assessed in the somatic whole-cell configuration of the patch-clamp technique, and diffusion of bis-Fura 2 into the apical dendrite (200–300 µm) took about 15 minutes. Recordings were done with an EPC10 patch-clamp amplifier (HEKA) controlled by PULSE software (HEKA). Within the first minute after establishing the whole-cell configuration the resting potential was directly measured (0 pA current injection). From the resting potential we then applied 900 ms long negative and positive current injections to examine passive membrane parameters and cellular excitability parameters, respectively (see below). Single somatic action potentials (APs) were elicited by brief current injections (4 ms) of variable strength. We injected increasing amounts of current (in steps of 15 pA) in order to catch the first action potential in response to a just suprathreshold current injection. This action potential was then further analyzed (see below).

### Ca^2+^ imaging experiments

To study action potential backpropagation in the apical dendrite of individual CA1 pyramidal cells we chose the Ca^2+^ imaging technique. This approach exploits the fact that backpropagating action potentials (b-APs) activate dendritic Cav channels which then mediate Ca^2+^ influx. The Ca^2+^ binds to and changes the fluorescence properties of a cytoplasmic dye (e.g. bis-Fura 2). This b-AP-induced Ca^2+^ signal is acquired and its amplitude measured at many different locations along the dendrite (see below). Unlike dendritic microelectrode recording, this method, which we refer to as “b-AP imaging”, is indirect and depends critically on the properties of the dendritic Cav channels. Also, the fluorescent dye represents a Ca^2+^ buffer which may modulate Ca^2+^-dependent processes in the cytoplasm. However, b-AP imaging also has several advantages [21]: Unlike rupturing the dendritic membrane with a microelectrode, b-AP imaging leaves the dendritic compartment largely unaffected, and no additional pipette capacitance is introduced, which may cause filtering of the recorded signals. The b-AP imaging technique also provides a high spacial resolution (see below), which can hardly be reached with multiple dendritic electrode recording from individual neurons. Finally, the success rate with the b-AP imaging technique is higher than with multiple dendritic electrode recording. Consequently, the number of cells included in a given data set is larger. This is a critical prerequisite for the interpretation of experiments with a large scatter of data points (see also Fig. S3).

For dendritic b-AP imaging runs we first adjusted the holding current to produce a stable membrane potential of -70 mV and then elicited single APs with a 1 nA somatic current injection of 4 ms duration. The fluorescence changes based on the b-AP-induced Ca^2+^ entry were monitored at a single excitation wavelength of 395 nm and a 91 Hz sequential frame-rate using a Polychrome V monochromator device controlled by TILLvisION Software (Till Photonics). Simultaneous movement of slice and patch-pipette in the whole-cell configuration with a shifting table (Luigs and Neumann) allowed successive b-AP imaging runs at high magnification (63 x) in more than one region of the dendritic tree. The 1002×1004 pixel arrays were symmetrically binned 8 times.

### Pharmacology

Control slices (from untreated animals) were studied under the influence of the Kv4 current activator N-[3,5-bis(trifluoromethyl)phenyl]-N′-[2,4-dibromo-6-(1H-tetrazol-5-yl)phenyl]-urea (NS5806, 20 µM) and the A-type channel inhibitor 4-aminopyridine (4-AP, 5 mM). The effects of both drugs were tested in the presence of the ionotropic glutamate receptor antagonists 6-cyano-7-nitroquinoxaline-2, 3-dione (CNQX, 10 µM) and DL-2-amino-5-phosphopentanoic acid (AP5, 50 µM). All drugs were purchased from Sigma-Aldrich, unless otherwise indicated, and were applied with a peristaltic pump-driven multi-channel bath perfusion system (ValveBank 8 II, AutoMate Scientific).

### Data analysis

The programs PULSEFIT (HEKA) and AxoGraph X (John Clements; http://axographx.com) were used to analyze the acquired voltage traces. We measured the resting potential and calculated membrane time constant and input resistance (R_in_). In addition, from the voltage sag seen with negative current injections the activation kinetics and magnitude of a hyperpolarization-activated current (*I*_h_) were estimated (see Fig. S1). From the traces obtained with a 50 pA positive current injection we determined AP latency and AP frequency. Individual APs were analyzed for the following parameters: threshold, amplitude (measured between peak and resting potential), upstroke velocity, halfwidth (width at 50% of the peak) and afterdepolarization (ADP; quantified as the integral below the corresponding part of the AP waveform and by the time constant of decay according to a single-exponential fit). Series resistance (R_s_)-related artefacts in the recorded voltage traces due to incomplete bridge balance were computed off-line and subtracted from the absolute voltage deflections measured.

TILLvisION Software (Till Photonics) was used to analyze b-AP imaging data in pre-defined regions of interest (ROIs) positioned in 10 µm intervals along the apical dendrite. B-AP imaging data from 3 runs with the same protocol were averaged for every ROI. Further off-line analysis was done with Excel (Microsoft). The data were corrected for bleaching by dividing the trace that included the Ca^2+^ signal by a pre-trace of the same length that included only linear bleaching. Relative changes in intracellular Ca^2+^ were quantified by calculating ΔF/F in %, where the baseline represents the fluorescence F, measured before current injection, and the change in fluorescence intensity ΔF represents the signal increase during the passage of a b-AP. Peak values of ΔF/F for individual ROIs at various locations along the apical dendrite were normalized to the corresponding first ROI (5 µm from the soma). The decay kinetics of the Ca^2+^ transient obtained next to the soma were approximated by a single-exponential fit.

Summary data are presented as mean ± standard error of the mean (SEM). Statistical analyses were done using InStat and Prism (GraphPad). We analyzed differences between cells from *sham*-treated animals (*sham* cells) and cells from kainate-injected animals with SE behavior (SE cells), as well as differences between parameters under control conditions and in the presence of a drug. Statistical significance of differences between the mean values of two groups of data (e.g. AP parameters of *sham* and SE cells, respectively) was tested with Sudent's unpaired *t*-test. Three independent groups of data (e.g. AP parameters of *sham* cells and two subdivisions of SE cells) were statistically analyzed by one-way analysis of variance (ANOVA) with Tukey's multiple comparison test. Differences in the propagation of the b-AP-induced Ca^2+^ signal were analyzed by comparing the slopes of linear regression lines, derived from logarithmized data (see Fig. S3). For a comparison of backpropagation in *sham* and SE cells Student's unpaired *t*-test was used. For a comparison of backpropagation under control conditions, in the presence of NS5806 and in the presence of 4-AP one-way ANOVA and Tukey's multiple comparison test were used. NS5806-induced changes in cell excitability and discharge behavior (see Fig. S4) were statistically analyzed with Student's paired *t*-test.

## Results

### Behavioral manifestation of kainate-induced status epilepticus in mice

After kainate injection the animals were monitored for 3–4 h to keep track of the behavioral manifestation of status epilepticus (SE, see Methods). In the present study we did not perform electroencephalographic recordings during SE induction [33], nor did we await recurrent seizures after the termination of a latent period as a clear indication of SE-related epileptogenesis. However, the observed time courses of behavioral stage transitions (SE profiles, see Methods and Fig. 1A) were in accord with previous descriptions of a rat kainate model [34]. From a total of 24 kainate-injected animals 15 (62.5%) showed drastic behavioral changes, with the first stage 5 seizures occurring within 59±3 min after kainate injection (Fig. 1B and C). The number of seizures ≥ stage 5 was 15±3 (n = 15; Fig. 1D), and the mean SE profile integral (area under the SE profile curve as a measure of SE severity, which also rates onset times and post-SE stages; see Methods) was 583±42 (n = 15; Fig. 1E). Taken together, the data documented a more or less uniform SE time course and severity for the 15 animals, which were used for further experimentation. Three animals (12.5%) showed sparse behavioral changes after kainate injection, and 6 animals (25.0%) died within 1–2 h after kainate injection (Fig. 1B). Animals from the two latter groups were not used for further experimentation.

### Acute changes in hippocampal CA1 pyramidal cell excitability following SE

**Figure 2:**
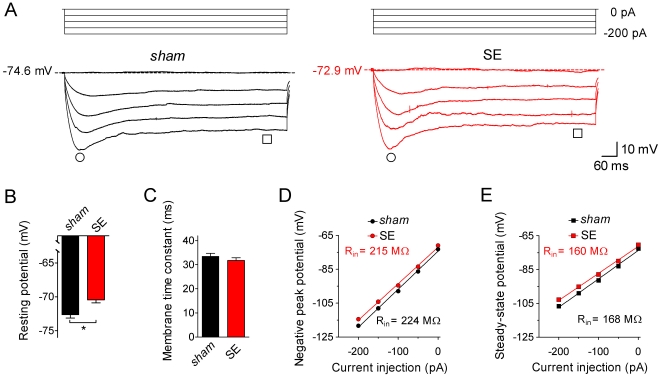
Subthreshold electrophysiological properties of CA1 pyramidal cells. Individual CA1 pyramidal cells in a hippocampal slice preparation were electrophysiologically characterized in the whole-cell patch-clamp configuration. **A**. Current-clamp recordings (test pulses of 0 to −200 pA for 900 ms as indicated) obtained from a CA1 pyramidal cell of a *sham*-treated animal (*sham*, black traces on the left) and from a CA1 pyramidal cell of a kainate-injected animal with SE behavior (SE, red traces on the right). Note that the potential measured with 0 pA current injection is slightly less negative in the SE cell. Time points for the measurement of negative peak potential (circles) and steady-state potential (squares) are indicated. **B**. Resting potential of *sham* (black bar, n = 65) and SE cells (red bar, n = 81); * p<0.05). **C**. Membrane time constant (obtained with a single-exponential fit to the initial voltage deflection in response to a −50 pA current injection) of *sham* (black bar, n = 65) and SE cells (red bar, n = 81). **D**. Negative peak potential (see circles in A) plotted against current injection for *sham* (black, n = 65) and SE cells (red, n = 81). **E**. Steady-state potential (see squares in A) plotted against current injection for *sham* (black, n = 65) and SE cells (red, n = 81); error bars in D and E are smaller than symbols; linear fits to the data yielded slopes from which input resistance (R_in_) was determined (numbers are mean values).

**Figure 3:**
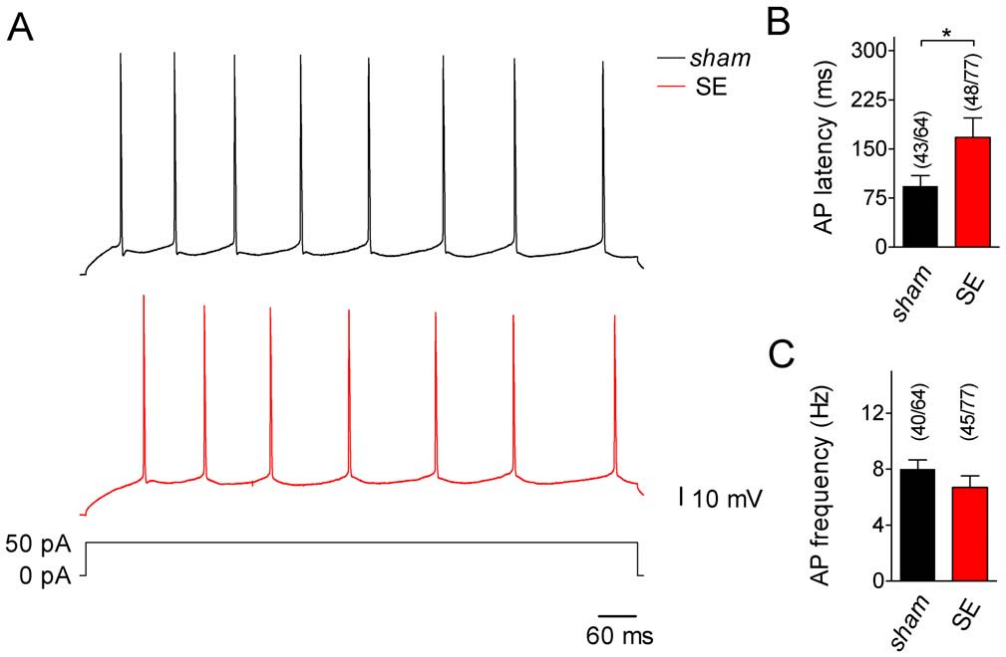
Excitability and discharge behavior of CA1 pyramidal cells. **A**. Current-clamp recordings (test pulse of +50 pA for 900 ms as indicated) from a *sham* (top, black trace) and an SE cell (bottom, red trace). Both cells show regular firing with no sign of burst behavior. Note, however, that in the SE cell it took longer than in the *sham* cell for the first action potential (AP) to appear. **B**. Time until the first AP appears (AP latency) in response to a +50 pA current injection for *sham* (black bar) and SE cells (red bar); fraction of cells in which firing threshold was reached with +50 pA is indicated (* p<0.05). **C**. AP frequency for *sham* (black bar) and SE cells (red bar); fraction of cells which fired repetitive APs at +50 pA is indicated.

**Figure 4:**
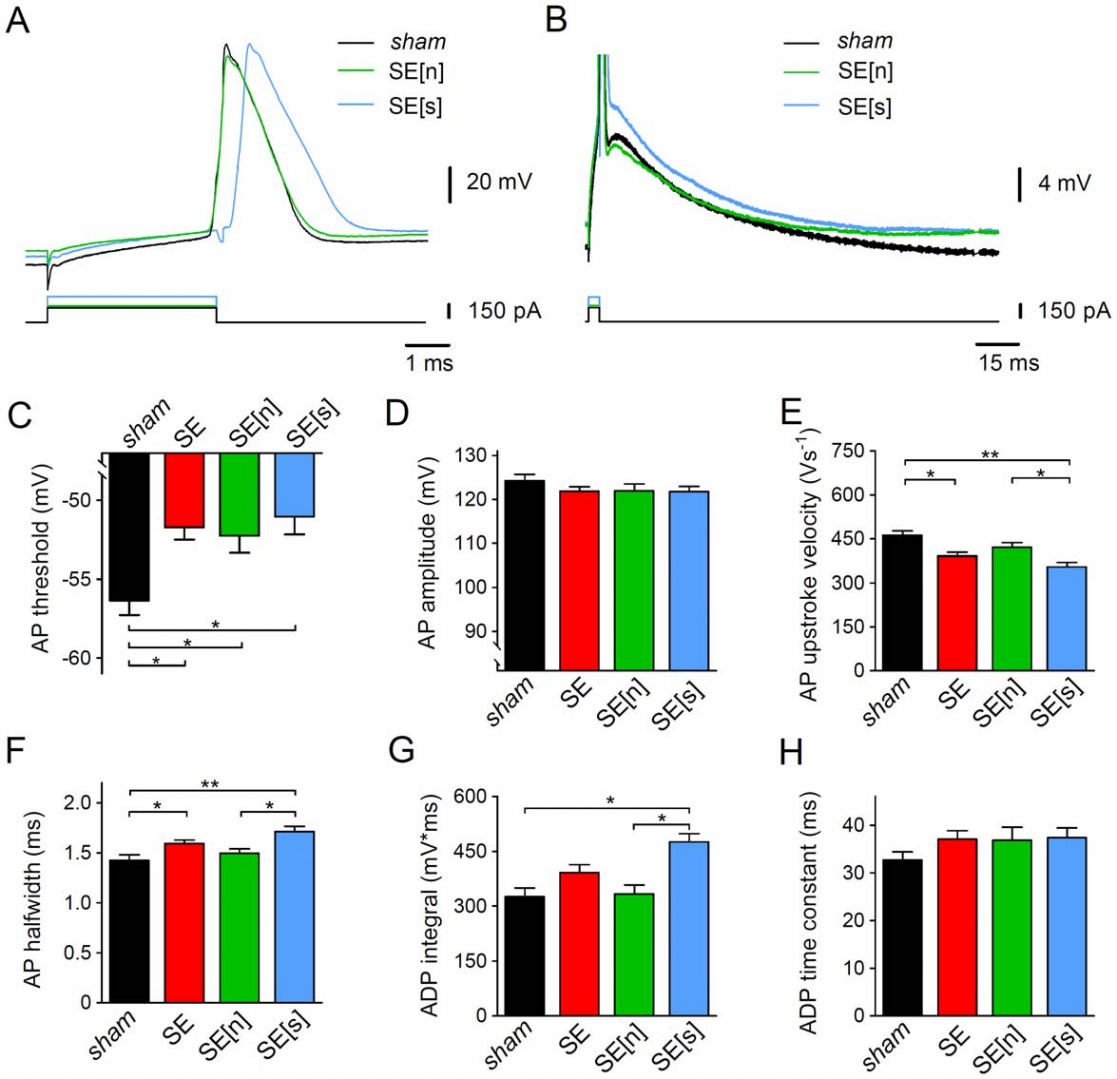
Properties of single somatic APs in CA1 pyramidal cells. **A** and **B**. Single somatic APs elicited by a just suprathreshold current injection of 4 ms duration (as indicated) in a *sham* cell (black traces) and different SE cells. SE cells were subdivided in SE[n] (notch present, green traces) and SE[s] cells (smooth, notch absent, blue traces). APs are shown on different time and voltage scales in A and B, respectively; panel A shows that in the SE cells the AP threshold was less negative, AP upstroke velocity smaller and AP halfwidth larger than in the *sham* cell (artefacts in the voltage traces coincide with the on- and offset of the current pulse but not with the AP peak. They were accounted for in the analysis, see Methods); panel B shows the presence (SE[n]) or absence (SE[s]) of the notch typically preceding after-depolarization (ADP) in *sham* cells. Compared to the *sham* cell, the area under the corresponding part of the voltage trace (ADP integral) was larger in the SE[s] cell. **C**. AP threshold for *sham* (black), SE (red), SE[n] (green) and SE[s] cells (blue); n = 37, 61, 34 and 27, respectively; **D**. AP amplitude for *sham*, SE, SE[n] and SE[s] cells (n = 37, 61, 34 and 27); **E**. AP upstroke velocity for *sham*, SE, SE[n] and SE[s] cells (n = 37, 61, 34 and 27); **F**. AP halfwidth for *sham*, SE, SE[n] and SE[s] cells (n = 37, 61, 34 and 27); **G**. ADP integral for *sham*, SE, SE[n] and SE[s] cells (n = 28, 34, 20 and 14); **H**. ADP time constant (of decay) for *sham*, SE, SE[n] and SE[s] cells (n = 28, 34, 20 and 14); * p<0.05; ** p<0.001 (ANOVA).

Immediately after the 3–4 h time interval of behavioral monitoring animals were sacrificed and hippocampal slices prepared. Individual CA1 pyramidal cells were electrophysiologically characterized in the whole-cell configuration in current-clamp mode (Fig. 2, 3, 4). These recordings revealed that some parameters of cell excitability differed significantly between CA1 pyramidal cells from *sham*-treated animals (*sham* cells) and CA1 pyramidal cells from kainate-injected animals that showed SE behavior (SE cells). At 0 pA current injection we recorded a resting potential of −72.6±0.5 mV (n = 65) in *sham* cells. In SE cells the resting potential was slightly less negative (−70.4±0.4 mV, n = 81, p = 0.001; Fig. 2A and B). To examine subthreshold membrane potential dynamics we applied negative current injections of 900 ms duration. These protocols resulted in hyperpolarization, which developed with a characteristic membrane time constant of 33.5±1.2 ms (n = 61) in *sham* and 32.2±1.1 ms (n = 71, p = 0.4083) in SE cells at a current injection of −50 pA (Fig. 2A and C). More negative current injections resulted in membrane hyperpolatization with a negative peak potential and a prominent sag (due to the activation of *I*_h_), which finally reached a steady-state potential (Fig. 2A). Both negative peak (Fig. 2D) and steady-state potential (Fig. 2E) were plotted against the injected current, and the respective slopes were analyzed to calculate the corresponding input resistances (R_in_; [11]). This analysis revealed similar R_in_ values for *sham* and SE cells (peak R_in_: 224±6 MΩ, n = 65 for *sham* and 215±5 MΩ, n = 81 for SE, p = 0.245; steady-state R_in_: 168±6 MΩ, n = 65 for *sham* and 160±5 MΩ, n = 81 for SE, p = 0.283; Fig. 2D and E). Also, neither the voltage sag amplitude (negative peak potential – steady-state potential), which represents a rough estimate of *I*_h_ magnitude, nor the sag time constant (reflecting *I*_h_ activation kinetics) were different in *sham* and SE cells (Fig. S1). These results suggest that acute SE caused a small but significant depolarization of the resting potential but had no effect on input resistance, membrane time constant and *I*_h_-related subthreshold membrane potential dynamics in hippocampal CA1 pyramidal cells.

To directly explore possible effects of acute SE on cell excitability we applied positive current injections of 50 pA (the approximate rheobase value determined for the cells under study; data not shown) and 900 ms duration. This protocol caused membrane depolarization, which resulted in the generation of an action potential (AP) or a series of APs (Fig. 3A) in 43 out of 64 *sham* cells (67%) and in 48 out of 77 SE cells (62%). Burst firing (as defined by Chen and coworkers [35]) was never observed in our experiments. Notably, the time it took for the (first) AP to appear (AP latency) differed between *sham* and SE cells. The AP latency was 93±16 ms (n = 43) in *sham* and 168±30 ms (n = 48, p = 0.035) in SE cells (Fig. 3B). On the other hand, AP frequency was not significantly different between *sham* (8.0±0.7 Hz, n = 40) and SE cells (6.7±0.8 Hz, n = 45, p = 0.230; Fig. 3C). These data suggest remodeling processes during acute SE that lead to a delay in AP firing at the rheobase level of current injection.

### Acute changes in action potential properties

To further analyze the effects of acute SE on CA1 pyramidal cell excitability and to explore possible effects on the properties of individual APs, we applied current-clamp protocols suited to elicit only a single AP. In particular, current injections of 4 ms duration were adjusted in strength for each cell to allow the recording of the AP response to a just suprathreshold current injection (244±14 pA, n = 37 in *sham* cells and 271±13 pA, n = 61 in SE cells, p = 0.1612; Fig. 4A and B). In *sham* cells the AP threshold was −56.4±0.9 mV (n = 37). In the SE cells the AP threshold was less negative (−51.7±0.8, n = 61, p = 0.0002; Fig. 4A and C). However, due to the concomitant positive shift in the resting potential the absolute voltage needed to bring the membrane potential from rest to threshold was not significantly different between *sham* (17.1±0.9 mV, n = 37) and SE cells (18.6±0.8 mV, n = 61, p>0.05, ANOVA; Fig. S2). Also the AP amplitude was not significantly different between *sham* (124.2±1.4 mV, n = 37) and SE cells (121.8±1.0 mV, n = 61, p = 0.1674; Fig. 4A and D).

The inspection of AP kinetics revealed significant differences between *sham* and SE cells. In *sham* cells AP upstroke velocity was 462±15 Vs^−1^ (n = 37), and AP halfwidth was 1.43±0.06 ms (n = 37). We found that AP upstroke velocity was lower (391±12 Vs^−1^, n = 61, p = 0.0004; Fig. 4A and E), and AP halfwidth was larger (1.59±0.04 ms, n = 61, p = 0.0299; Fig. 4A and F) in SE cells. No significant differences between *sham* and SE cells were found with respect to afterdepolarization (ADP) integral (*sham*: 326±24 mV*ms, n = 28; SE: 392±21 mV*ms, n = 34, p = 0.0992; Fig. 4G) and the corresponding ADP time constant of decay (*sham*: 32.7±1.8 ms, n = 28; SE: 37.1±1.8 ms, n = 34, p = 0.0891; Fig. 4H).

Notably, a fraction of SE cells (27 out of 61 cells; 44%) did not show the notch, which typically precedes ADP in CA1 pyramidal cells and which was seen in all 37 *sham* cells tested (Fig. 4B). The finding that the AP notch was absent in a fraction of SE cells prompted us to statistically compare the above parameters for the subgroups SE[n] (notch) and SE[s] (smooth due to absence of notch) of the parent population of all SE cells (ANOVA, see Methods and Fig. 4C–H). This analysis revealed that the difference found between *sham* and SE for AP threshold was not affected by the subdivision in SE[n] and SE[s] (Fig. 4C). The voltage needed to bring the membrane potential from rest to threshold (Fig. S2) as well as the AP amplitude (Fig. 4D) were not significantly different among all groups. By contrast, only in SE[s] but not in SE[n] cells was the AP upstroke velocity and AP halfwidth significantly different from the corresponding values in *sham* cells, and SE[s] cells had a significantly larger AP upstroke velocity and AP halfwidth than SE[n] cells (Fig. 4E and F). The ADP integral in SE[s] cells was significantly different from both *sham* and SE[n] (Fig. 4G), whereas the ADP time constant was not significantly different among all groups (Fig. 4H). These results suggest that acute SE caused a depolarizing shift in AP threshold and a reduction of AP upstroke velocity. They further suggest that in some of the tested neurons acute SE abolished the notch, which normally precedes ADP, and led to spike broadening and augmentation of ADP.

### Acute changes in dendritc spread of excitation

**Figure 5:**
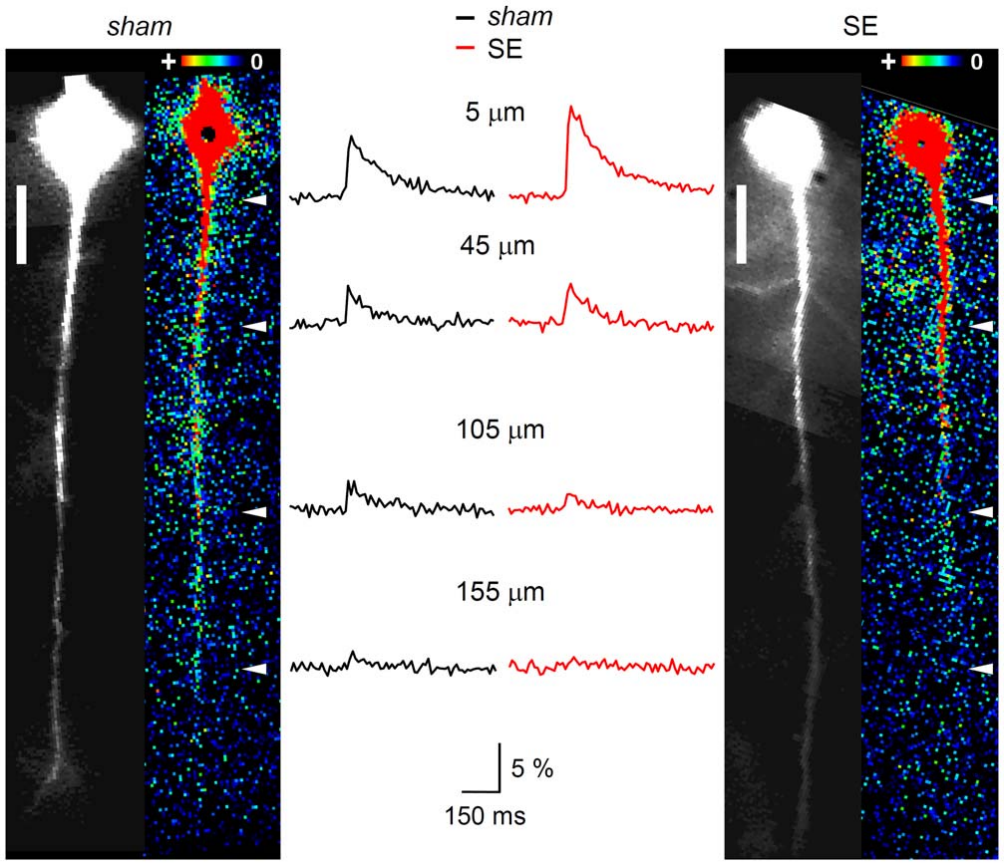
B-AP imaging in the apical dendrite of CA1 pyramidal cells. The retrograde spread of excitation in the apical dendrite of CA1 pyramidal cells was studied with the Ca^2+^ imaging technique. **A** and **C**, left (black-and-white images). *Sham* and SE cell, respectively, filled with bis-Fura 2 via a somatic patch-pipette (not shown); scale bars: 25 µm. **A** and **C**, right (false color images). Same *sham* and SE cell, respectively, during backpropagation of an AP, which was generated by somatic current injection. The maximal Ca^2+^ signal (absolute fluorescence change, ΔF) caused by the b-AP along the apical dendrite is color-coded (see insets): red (+) represents large, dark blue low changes in fluorescence. **B**. Corresponding Ca^2+^ transients (ΔF/F in %) recorded from the *sham* (black) and the SE cell (red) shown in A and C, respectively, at 5, 45, 105 and 155 µm from the soma (see white arrowheads in A and C, right). Note that, despite the larger amplitude of the Ca^2+^ signal next to the soma, its decrease along the apical dendrite was stronger in the SE cell than in the *sham* cell.

**Figure 6:**
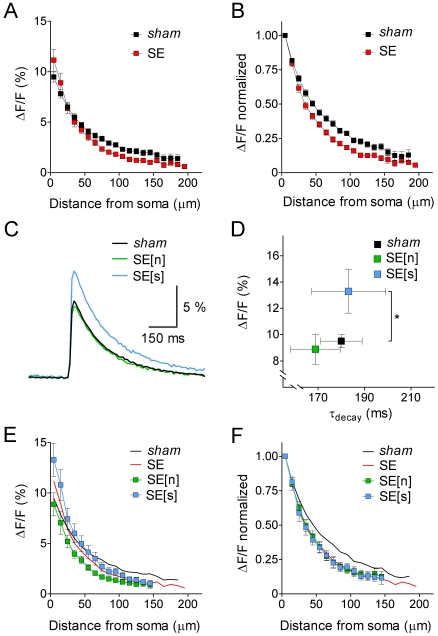
Quantitative analysis of the dynamics of b-AP-induced Ca^2+^ signals in CA1 pyramidal cells. **A**. Maximal fluorescence changes (ΔF/F in %) along the apical dendrite (10 µm steps, starting 5 µm from the soma) for *sham* (black, n = 41 continuous data sets averaged) and SE cells (red, n = 39 continuous data sets averaged). Ca^2+^ signals in the proximal dendrite were slightly larger, but the overall attenuation of the b-AP-induced Ca^2+^ signal along the apical dendrite was stronger in SE versus *sham* cells (cross-over of data sets at 25 µm from the soma). **B**. B-AP imaging data normalized to the signal next to the soma for *sham* (black, n = 41) and SE cells (red, n = 39). **C**. Averaged Ca^2+^ signal traces next to the soma for *sham* (black, n = 41), SE[n] (green, n = 17) and SE[s] cells (blue, n = 12). **D**. Comparison of Ca^2+^ signal amplitude and decay kinetics measured next to the soma for *sham* (black, n = 41), SE[n] (green, n = 17) and SE[s] (blue, n = 12); * p<0.05. **E**. B-AP analysis (ΔF/F in % along the apical dendrite) for SE[n] (green, n = 17) and SE[s] cells (blue, n = 12); lines without symbols represent corresponding data from *sham* (black) and the parent population of SE cells (red; see panel A). **F**. Normalized b-AP imaging data for SE[n] (green, n = 17) and SE[s] cells (blue, n = 12); lines without symbols represent corresponding data from *sham* (black) and the parent population of SE cells (red; see panel B). Note that SE[n] and SE[s] data superimpose with parent SE data when normalized.

Next we asked whether the backpropagation of individual APs in the apical dendrite of hippocampal CA1 pyramidal cells was influenced by acute SE. To answer this question we elicited single APs at the soma and monitored the propagation of the b-AP-induced Ca^2+^ signal (see Methods) in *sham* and SE cells. Figure 5 shows b-AP imaging experiments performed on a *sham* cell (Fig. 5A) and on an SE cell (Fig. 5C). It can be seen that dye-filling of the neurons via the patch-pipette resulted in a diffusion gradient of bis-Fura 2 with massive loading of the soma and a lower but clearly detectable baseline fluorescence in distal portions of the apical dendrite (200 µm from the soma; black-and-white images in Fig. 5A and C, left). The b-AP-triggered Ca^2+^ influx is illustrated as absolute fluorescence change (ΔF) in false color images (Fig. 5A and C, right). It can be anticipated that in the *sham* cell (Fig. 5A, right) the Ca^2+^ signal strongly decreased after the first 50 µm of the apical dendrite and finally vanished at a distance of about 175 µm from the soma. In the SE cell (Fig. 5C, right) the Ca^2+^ signal apparently showed a strong decrease beyond 75 µm from the soma. Notably, however, in the SE cell the Ca^2+^ signal abruptly disappeared at a distance of 125 µm from the soma. The actual Ca^2+^ transients (relative fluorescence change ΔF/F over time) measured at four different locations along the apical dendrite (between 5 and 155 µm from the soma, as indicated in Fig. 5A and C, right) are shown in Figure 5B. In the *sham* cell (Fig. 5B, left) the b-AP caused a Ca^2+^ signal, which was reduced to about one fourth of its original size at the most distal location (Fig. 5B, left). In the SE cell (Fig. 5B, right) the amplitude of the Ca^2+^ signal next to the soma (5 µm distance) was one and a half times larger than the Ca^2+^ signal of the *sham* cell at the same location. However, at the most distal location the reduction was stronger in the SE cell, and the Ca^2+^ signal was below the detection level (Fig. 5B, right). Figure 6A shows the peak amplitudes of the b-AP-induced Ca^2+^ signals (ΔF/F) along the apical dendrite averaged for all *sham* and SE cells tested. It can be seen that, compared to *sham*, the average Ca^2+^ signal of SE cells was higher next to the soma but more strongly suppressed in distal portions of the dendrite. In *sham* cells the signal amplitude (ΔF/F) decreased from 9.5±0.5% (n = 41) next to the soma to 1.4±0.4% (n = 4) at a distance of 185 µm, and in SE cells from 11.1±1.1% (n = 39) next to the soma to 0.6% (n = 1) at a distance of 195 µm from the soma. The cross-over of the two data sets occurs at a distance of about 25 µm from the soma. For a better quantification of putative effects of acute SE on the attenuation of the b-AP-induced Ca^2+^ signal the peak amplitudes obtained at different locations along the apical dendrite were normalized to the value obtained next to the soma for each individual cell, and the results were averaged for *sham* and SE. The corresponding summary data are shown in Figure 6B. It can be seen that the attenuation of the b-AP-induced Ca^2+^ signal was strengthened following acute SE (see also Fig. S3).

The larger Ca^2+^ signals observed in SE cells at locations within 25 µm from the soma may be explained by SE-induced spike broadening (increase in AP halfwidth, see Fig. 4F) and ADP augmentation (increase in ADP integral, see Fig. 4G), which may prolong the AP-induced Ca^2+^ influx. Since AP halfwidth and ADP integral critically depended on the absence or presence of the AP notch in SE cells (see Fig. 4F and G) we once more divided the SE parent population in SE[n] and SE[s] cells for a separate analysis. Figure 6C shows averaged Ca^2+^ traces for *sham*, SE[n] and SE[s], respectively, measured next to the soma. The peak amplitude of the SE[n] signal (8.9±1.1%, n = 17) was not significantly different from *sham* (9.5±0.5%, n = 41; p>0.05), whereas the peak amplitude of the SE[s] signal was much larger (13.3±1.7%, n = 12; p<0.05; Fig. 6C and D). Notably, the decay time constants of the Ca^2+^ transients were not significantly different among the three groups (*sham*: 180±9 ms, n = 41; SE[n]: 169±11 ms, n = 17; SE[s]: 184±16 ms, n = 12; p>0.05; Fig. 6D). To test whether SE[n] and SE[s] cells made an equal contribution to the observed average strengthening of the attenuation of the b-AP-induced Ca^2+^ signal after acute SE, we analyzed the respective b-AP imaging data separately (Fig. 6E and F). Figure 6E shows that, although the SE[s] data start-off at a larger signal amplitude next to the soma, they clearly undercut the *sham* data more distally (cross-over at about 60 µm from the soma) and eventually align with SE[n] data (at 150 µm from the soma). Moreover, when all b-AP imaging data sets were normalized to the values obtained next to the soma it became evident that both SE[n] and SE[s] data align with the data from the undivided SE parent population (Fig. 6F). Thus, despite different signal amplitudes next to the soma, SE[n] and SE[s] cells make an equal contribution to the observed strengthening of the attenuation of the b-AP-induced Ca^2+^ signal along the apical dendrite. Taken together, the analysis of our b-AP imaging data suggests that acute SE led to a stronger than normal b-AP attenuation. The molecular and/or cellular remodeling processes, which may underlie the strengthening of b-AP attenuation, are unrelated to the mechanism(s) leading to spike broadening and ADP augmentation.

### Pharmacological modulation of the dynamics of b-AP-induced Ca^2+^ signals in hippocampal CA1 pyramidal cells

**Figure 7:**
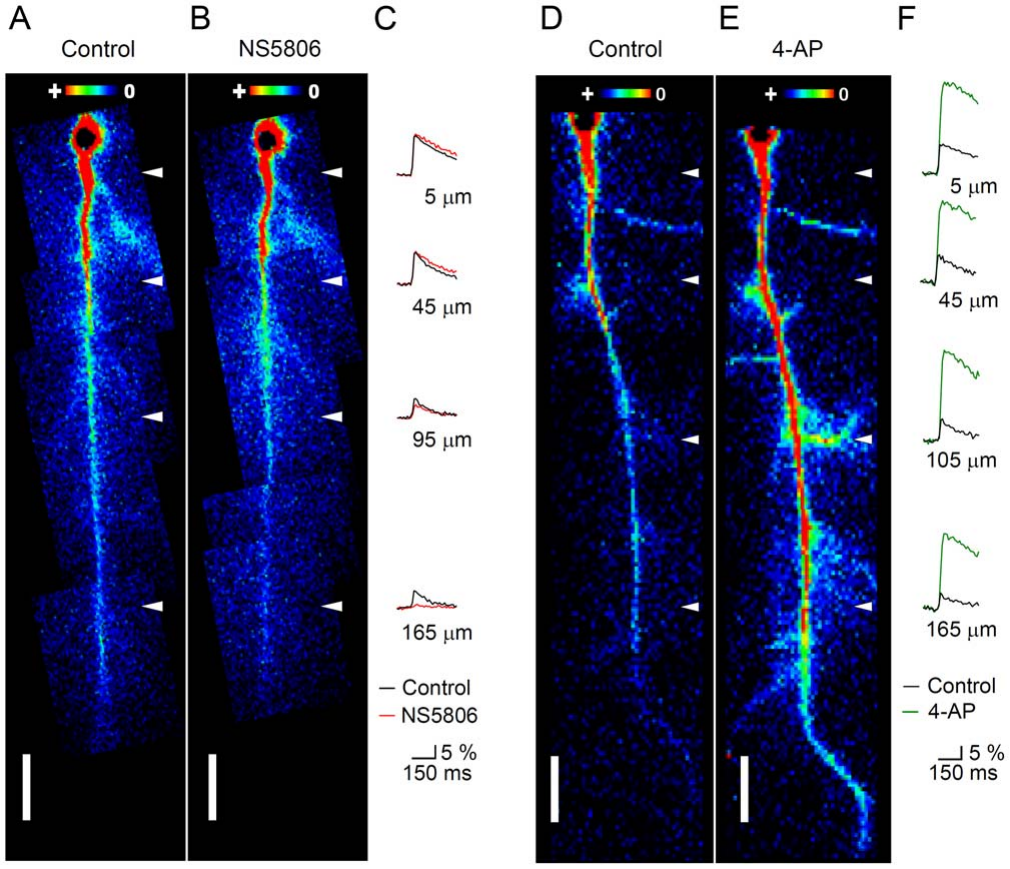
Effect of NS5806 and 4-AP on the dynamics of b-AP-induced Ca^2+^ signals. **A** and **B**. False color images of a CA1 pyramidal cell from an untreated animal in the absence (Control) and presence of 20 µM NS5806, respectively, during AP backpropagation. The maximal Ca^2+^ signal (absolute fluorescence change, ΔF) caused by the b-AP is color-coded (insets). **C**. Corresponding Ca^2+^ transients (ΔF/F in %) recorded at 5, 45, 95 and 165 µm from the soma (see white arrowheads in A and B) in the absence (black traces) and presence of NS5806 (red traces). Note that the Ca^2+^ signal at 165 µm from the soma was almost below the detection level in the presence of NS5806. **D** and **E**. CA1 pyramidal cell in the absence (Control) and presence of 5 mM 4-AP, respectively, during AP backpropagation. Note that the Ca^2+^ signal was still visible in the most distal parts of the apical dendrite and invaded lateral branches in the presence of 4-AP. **F**. Corresponding Ca^2+^ transients recorded at 5, 45, 105 and 165 µm from the soma (see white arrowheads in D and E) in the absence (black traces) and presence of 4-AP (green traces). Note that the Ca^2+^ signal showed only little attenuation along the apical dendrite in the presence of 4-AP.

**Figure 8:**
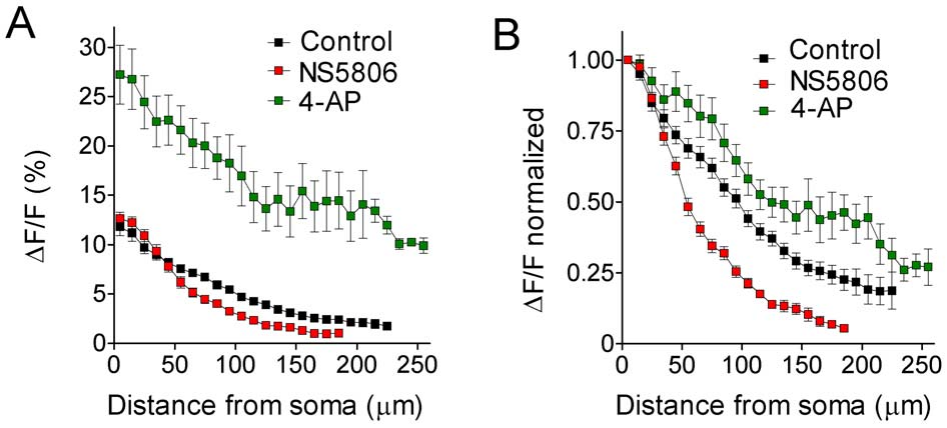
Analysis of pharmacological modulation of b-AP-related Ca^2+^ signal dynamics. **A**. Maximal fluorescence changes (ΔF/F in %) along the apical dendrite (10 µm steps, starting 5 µm from the soma) under control conditions (black; n = 36 continuous data sets averaged), in the presence of NS5806 (red; n = 15 continuous data sets averaged) and in the presence of 4-AP (green; n = 15 continuous data sets averaged). **B**. B-AP imaging data normalized to the signal next to the soma for control (black, n = 36), NS5806 (red, n = 15) and 4-AP (green, n = 15). Note that the attenuation of the b-AP-induced Ca^2+^ signal was strengthened in the presence of NS5806 and weakened in the presence of 4-AP.

The observed strengthening of the attenuation of the b-AP-induced Ca^2+^ signal following acute SE may be explained by an increased activity of a subthreshold-activating somatodendritic A-type K^+^ current (*I*_SA_). Since *I*_SA_ is mediated mainly by Kv4 channels [36]–[38], we tested whether the previously described Kv4 current activator N-[3,5-bis(trifluoromethyl)phenyl]-N′-[2,4-dibromo-6-(1H-tetrazol-5-yl)phenyl]-urea (NS5806, [39]) was able to reproduce part of our SE results. Furthermore, as a negative control, we tested whether the A-type K^+^ channel inhibitor 4-aminopyridine (4-AP) did the contrary. We studied the effects of the two drugs on the propagation of the b-AP-induced Ca^2+^ signal in CA1 pyramidal cells in hippocampal slices obtained from untreated (neither *sham* nor kainate-injected) animals (see Methods). Figure 7 shows b-AP imaging experiments performed on a cell in the absence and presence of 20 µM NS5806 and on a cell in the absence and presence of 5 mM 4-AP. Under control conditions the Ca^2+^ signal gradually declined along the apical dendrite and finally vanished at around 200 µm from the soma (Fig. 7A and D). When NS5806 was applied the decline of the Ca^2+^ signal was considerably stronger and the signal vanished already at a shorter distance from the soma (Fig. 7B). Figure 7C shows the corresponding Ca^2+^ transients (in the absence and presence of NS5806) at four different locations along the apical dendrite (as indicated in Fig. 7A and B): next to the soma NS5806 caused a slight increase of the Ca^2+^ signal. However, at the most distal location, where the signal was about half of its original size under control conditions, the reduction was much stronger, and the Ca^2+^ signal was almost below the detection level in the presence of NS5806 (Fig. 7C). Bath application of 4-AP largely prevented the decline of the Ca^2+^ signal. The spread of the signal now reached more distal parts of the dendrite and was still prominent 280 µm from the soma (Fig. 7E). Also, in the presence of 4-AP the Ca^2+^ signal invaded lateral branches of the apical dendrite (Fig. 7E). The Ca^2+^ transients at four different locations along the apical dendrite in the absence and presence of 4-AP are shown in Figure 7F: In the presence of 4-AP the Ca^2+^ signal next to the soma was about 3 times larger than under control conditions and did not show much reduction at the most distal location. Figure 8A shows the averaged peak amplitudes of the b-AP-induced Ca^2+^ signal along the apical dendrite under control conditions, in the presence of NS5806 and in the presence of 4-AP. It can be seen that, relative to control, NS5806 reduced the maximal range for Ca^2+^ signal detection, whereas 4-AP allowed the detection of Ca^2+^ signals still at larger distances (more than 250 µm) from the soma. Under control conditions the signal amplitude (ΔF/F) declined from 11.6±0.9% (n = 36) next to the soma to 1.8±0.4% (n = 6) at a distance of 225 µm, and with NS5806 from 12.6±0.7% (n = 15) next to the soma to 1.1±0.01% (n = 2) at a distance of 185 µm. 4-AP caused a dramatic increase in the mean signal amplitude at all locations along the dendrite, with 27.2±3.0% (n = 15) next to the soma and 9.9±0.8% (n = 2) at a distance of 255 µm (Fig. 8A). The normalized summary data are shown in Figure 8B. A clear strengthening of the normal attenuation of the b-AP-induced Ca^2+^ signal, especially within a range of 50–120 µm from the soma, can be observed in the presence of NS5806, whereas 4-AP does the contrary (Fig. 8B, see also Fig. S3). These data suggest that NS5806 helps to dampen the retrograde spread of excitation in the dendrites of hippocampal CA1 pyramidal cells. The effects of NS5806 on the dynamics of b-AP-induced Ca^2+^ signals were very similar to the ones seen after acute SE (see Fig. S3). To further explore the effects of NS5806 on CA1 pyramidal cell physiology we tested whether NS5806 had any influence on excitability and individual AP properties. We observed that NS5806 left membrane time constant and R_in_ unaffected while reducing AP upstroke velocity and increasing AP halfwidth (Fig. S4), similar to acute SE. Unlike acute SE, NS5806 caused an increase in AP frequency, a decrease in AP amplitude with no effect on AP threshold and a decrease in ADP integral (Fig. S4).

Taken together, our results suggest multiple mechanisms of remodeling in hippocampal CA1 pyramidal cells, which are already active during acute SE. These remodeling processes may lead to reduced excitability and, despite a concomitant spike broadening, to a strengthening of the attenuation of b-AP-induced Ca^2+^ signals, which is mimicked by the drug NS5806.

## Discussion

We have studied the excitability, AP waveform and somatodendritic spread of excitation in hippocampal CA1 pyramidal cells immediately after acute SE in mice, to test for cellular correlates of intrinsic plasticity before or at an early stage of epileptogenesis. Our data suggest that multiple remodeling processes may be active during acute SE. In particular, we observed a reduced excitability, a modulation of the AP waveform and a strengthened attenuation of b-AP-induced dendritic Ca^2+^ signals. In the absence of direct functional and molecular data on somatodendritic ion channel expression the mechanistic interpretation of our results must remain in part speculative.

### Cellular excitability and action potential firing after SE

Our experimental results show that already within a 1–2 h period after kainate injection excitability parameters of hippocampal CA1 pyramidal cells may be modulated. The observed changes are small on average but may be of pathophysiological relevance. The data support the notion that excitability and spread of dendritic excitation is reduced at this initial time point. Our results are unlikely to reflect a long-lasting form of postictal depression. This form of depression of cellular excitability normally lasts for a few minutes after the termination of a seizure and is thought to be mainly due to ionic dynamics changes including elevated internal Na^+^ and lowered external K^+^ [40]. A significant contribution of the electrogenic action of the Na^+^/K^+^ ATPase, stimulated under these conditions and supporting membrane hyperpolarization, has also been considered [40]. During our whole-cell experiments performed in slice preparations 1–5 h after brain isolation ionic conditions are expected to be dictated by the composition of the experimental solutions (see Methods). Also, our data suggest SE-induced depolarization rather than hyperpolarization of the resting potential. Therefore, we consider it more likely that intrinsic plasticity processes underlie the changes in cellular excitability.

The observed depolarization of the resting potential in CA1 pyramidal cells may be explained by downregulation of a resting K^+^ leak conductance via a massive activation of metabotropic glutamate receptors [41] during kainate-induced SE. If so, one would expect a concomitant increase in input resistance (R_in_). However, in our experiments SE caused a non-significant trend towards lower rather than higher R_in_ values. Alternatively, upregulation of a voltage-dependent Na^+^ conductance, tonically active around −70 mV, like *I*_h_, may underlie the slight depolarization of the resting potential. The data on SE-related *I*_h_ modulation in the literature are diverse: *I*_h_ upregulation in the hippocampus has been demonstrated 2 weeks after hyperthermic seizure induction [42]. Another study has reported upregulation of *I*_h_ 1–2 days and downregulation of *I*_h_ 4 weeks after kainate SE [43]. But *I*_h_ has also been shown to be downregulated as early as 24 h after pilocarpine SE in the entorhinal cortex [44]. We have not directly measured *I*_h_, but our voltage sag measurements do not support acute SE-related *I*_h_ modulation (see Fig. S1). Since we were interested in correlates of intrinsic plasticity we did not address acute remodeling of GABA-mediated inhibition [45] as a possible mechanism underlying the depolarization of the resting potential.

The positive shift in AP threshold and reduced AP upstroke velocity after acute SE suggest intrinsic plasticity processes related to Nav channel density and/or properties. It should be noted that in chronic epilepsy models no change in AP threshold has been reported [27], [46], [47], although Nav channels seem to undergo functional [48], [49] as well as transcriptional remodeling [50]. A downregulation of Nav1.6 mRNA, as reported for dentate granule cells one day after pilocarpine-induced SE [50], would be a likely explanation for the changes in the triggering and build-up of individual APs observed after acute SE in the present study.

The prolongation of AP latency observed after acute SE cannot be explained by the positive shift in AP threshold, since, due to the concomitant shift of the resting potential, the absolute voltage needed to bring the membrane from rest to threshold remained unchanged (see Fig. S2). It might be that upregulation of a subthreshold active K^+^ channel conductance, like *I*_D_ or *I*_SA_ [51], is involved in the delay of AP firing. According results obtained after overexpression of Kv4.2, the molecular substrate of *I*_SA_ [36]–[38], in hippocampal CA1 pyramidal cells [11] support this notion.

Overexpression of Kv4.2 has also been reported to cause faster spike repolarization [11], whereas in our experiments SE-related spike broadening was observed. The observed spike broadening was tightly coupled to the loss of the AP notch (see Fig. 4), and may therefore be related to downregulation of a K^+^ conductance distinct from *I*_SA_. BK-mediated currents, which normally cause fast notch-like re- and hyperpolarization [12], [14], would be a possible candidate. In fact, downregulation of hippocampal BK channel mRNA 24 h after pilocarpine SE [52] as well as in the chronic disease state [53] has been reported. BK channel function has also been shown to be upregulated in neocortical neurons 24 h after picrotoxin-induced seizures with a concomitant increase in firing frequency [54]. However, in the present study we never observed that acute SE caused increased firing frequency or bursting behavior in CA1 pyramidal cells, as often described in the chronic models [27]. Burst firing is supported by an upregulation of a persistent Na^+^ current [35] and Cav3.2-mediated LVA Ca^2+^ currents [55]–[57]. These conductances are majorly responsible for a pathophysiological augmentation of ADP [4]. In our experiments we also observed an SE-related increase in ADP integral, however, since this ADP augmentation was tightly coupled to the loss of the AP notch, it may not have been directly mediated by a remodeling of Na^+^ and Ca^2+^ conductances. A more indirect modulation of ADP in our experiments, possibly by BK channel modulation (see above), is also supported by the finding that ADP decay kinetics were not affected by acute SE.

It should be noted that our recordings were all done in the whole-cell configuration for the purpose of dye-filling. The inevitable wash-out of cytoplasmic components, including ion channel modifying enzymes and second messengers, in this recording configuration and the introduction of an exogenous Ca^2+^ buffer in the form of bis-Fura 2 may have masked SE-related changes in firing behavior. Also, the fact that our recordings were done at room temperature may have precluded burst firing. Nevertheless, our experimental results reveal differences in somatic excitability and individual AP properties between cells that had experienced SE and seizure navïe *sham* cells. These differences are an indication of intrinsic plasticity during acute SE.

### Activity-dependent remodeling of dendritic excitability

Dendrites are highly specialized and delicate structures, which control the input-output relations of neurons [58]. Dendritic membranes are able to generate APs (dendritic spikes) in response to excitatory synaptic input [59], and APs generated at the soma may be actively propagated, in a retrograde manner, along the apical dendrite [20], [21]. The dendritic APs are carried by a combination of voltage-dependent Na^+^ and Ca^2+^ currents [59]. It is not exactly known what the relative contribution of the respective Nav and different types of Cav channels to dendritic AP generation and propagation is, depending on the location along the apical dendrite. Immunogold staining suggests a negative gradient with distance from the soma for Nav1.6 in CA1 pyramidal cell dendrites [60]. But on the other hand, single channel recordings support a more or less uniform channel distribution for both Nav and Cav channels, albeit LVA Ca^2+^ channels display an apparent increase in density with distance from the soma [61]. Both the generation of dendritic spikes and AP backpropagation are limited by *I*_SA_, which is mediated by Kv4.2 channels in CA1 pyramidal cells [62]. The increase in *I*_SA_ amplitude with distance from the soma is thought to be primarily responsible for the strong b-AP attenuation seen in CA1 pyramidal cells along the apical dendrite [22].

Molecular remodeling processes in the dendrite may be critically involved in epileptogenesis [63]. In principle any of the aforementioned major dendritic ion channel conductances (Nav, Cav and Kv) may be modulated in an activity-dependent manner, leading to measurable intrinsic plasticity in the dendrite. A non-uniform remodeling of Nav and/or Cav channel density along the apical dendrite may underly our experimental results. Ellerkmann and coworkers [50] have shown SE-related downregulation of Nav1.6 in dentate granule cells. A similar downregulation in CA1 pyramidal cells may also lead to stronger than normal b-AP decline along the apical dendrite and would be in accordance with the observed changes in single AP properties (see above). Of course, downregulation of Cav channel function would inevitably lead to an attenuation of the Ca^2+^ signal detected in our imaging experiments. Notably, however, our data show that the Ca^2+^ signal is increased rather than decreased at the soma, in accordance with a previously reported SE-related upregulation [55], [56] rather than a downregulation of Cav channels.

*I*_SA_ may also be subject to significant remodeling with consequences for the dendritic spread of ecxitation. Precedent work in this respect stems from Bernard and coworkers [28], who tested b-AP attenuation in a pilocarpine model of acquired epilepsy in rats. It was found that in the chronic phase of the disease model b-AP attenuation in the distal portion of the apical dendrite was weaker than normal; i.e. the amplitude showed a normal decline up to a distance of 200 µm from the soma but was not further reduced as the AP traveled beyond that distance [28]. Downregulation of *I*_SA_-mediating Kv4.2 channels was made responsible for this chronic form of b-AP modulation [28], and in support of this notion, the authors found a decrease in total Kv4.2 protein and an increase in the fraction of extracellular receptor kinase (ERK)-phosphorylated Kv4.2 protein [28]. Kv4.2 downregulation in the chronic disease state of this model was also supported by immunocytochemistry experiments [64].

Weakened b-AP attenuation in the dendrite due to a chronic SE-related downregulation of Kv4.2 channels represents an acquired channelopathy [28]. To assess the causal relationship between altered b-AP dynamics and epileptogenesis it is important to determine the time of onset of changes in b-AP dynamics. In a first attempt to answer this question, we performed b-AP imaging experiments on individual CA1 pyramidal cells immediately after a 1–2 h period of kainate-induced SE in mice. Although the measurements performed in the present study are indirect (see Methods), our results suggest a strengthening of b-AP attenuation between 5 and 200 µm from the soma, a range where no difference between SE and control had been seen previously in the chronic phase of the rat pilocarpine model [28]. It should be noted that the range of measurement in our study was smaller than in the study by Bernard and coworkers [28], and we have no information about b-AP dynamics beyond 200 µm from the soma, the dendritic region, where drastic differences had been seen in the chronic pilocarpine model both with respect to b-AP suppression [28] and with respect to EPSP generation mediated by direct input from the entorhinal cortex ([65]; see below). In our experiments the suppression of the b-AP-induced Ca^2+^ signal was almost complete at the largest distance measured, and an increase in amplitude beyond a distance of 200 µm from the soma seems unlikely. Assuming that both before and during epileptogenesis *I*_SA_ governs b-AP dynamics, our b-AP imaging results may be explained by an augmentation of *I*_SA_.

There is experimental evidence of acute and subacute (24 h) SE-related Kv4.2 channel remodeling, however, the data reported in the literature are diverse: Su and coworkers [66] have tested protein expression levels in the rat pilocarpine model. They found that Kv4.2 protein and Kv channel interacting protein 1, a β-subunit of Kv4.2, were transiently upregulated in a seizure-dependent manner between 3 and 24 h after SE induction. These authors also measured 4-AP-induced Ca^2+^ elevations in CA1 pyramidal cells and reported a 2-fold larger rise in the SE group compared to control 24 h after pilocarpine injection. This finding was interpreted as an SE-related upregulation of functional Kv4.2 channels to be targeted by 4-AP [66]. The results also supported a previous report on hippocampal subfield-dependent remodeling, including an upregulation of Kv4.2 gene expression in the dentate granule cell layer, 24 h after a 5 min episode of kainate-induced seizures in rats [67]. Intriguingly, Kv4.2 gene expression in dentate granule cells has initially been reported to be transiently downregulated 3–6 h after metrazole-induced seizure activity in rats and to recover back to normal levels after 24 h [68]. Lugo and coworkers [33] have shown that an increase in ERK activity correlates with an increase in ERK-phosphorylated Kv4.2 in CA1, CA3 and dentate gyrus as early as 1 h after kainate-induced SE in rats, with a steady-state being reached between 1 and 3 h after kainate injection. The Kv4.2 content in a synaptosomal preparation was found to be decreased, whereas total Kv4.2 protein was found to be unchanged 3 h after SE induction by these authors [33]. Functional data documenting the generation of APs and dendritic spread of excitation at an early time point after SE have not been available so far. The results of the present study suggest a strengthening of b-AP attenuation after acute SE. Whether Kv4.2 remodeling plays a central role in this early form of intrinsic plasticity is unclear at present.

Our data support the notion that already during acute SE many remodeling processes related to intrinsic plasticity in CA1 pyramidal cells take place. The experimental results may be explained by a combination of Nav current modulation and *I*_SA_ modulation. However, these scenarios remain speculative, and the molecular mechanisms, which underlie acute SE-related intrinsic plasticity in CA1 pyramidal cells, still need to be identified.

### Experimental investigation and prevention of epileptogenesis

The process of epileptogenesis and the chronic state of epilepsy have been experimentally investigated in a variety of animal models (see examples above and [69]). A general problem in this context is that different animal models show some variability in their ethiology depending on the mode of seizure induction (e.g., kindling, pilocarpine or kaiante). Moreover, the use of different species (rat or mouse) and different ages of experimental animals (juvenile or adult) makes a comparison of the results obtained in different studies difficult. Despite these obvious limitations, animal models are necessary and may be useful to elucidate common mechanisms of epileptogenesis and to identify molecular targets in order to eventually explore the possibilities of antiepileptogenic treatment.

In the present study systemic kainate injection was used to induce SE in juvenile mice. Kainate excites many different types of neurons, including inhibitory interneurons, in a variety of brain regions, but CA3 pyramidal cells, the hippocampal pacemaker for the generation of synchronized activity to be propagated to CA1, are amongst the most responsive neurons to kainate in the brain [24]. This is due to the presence of a high affinity kainate receptor subtype on CA3 pyramidal cells in the mossy fiber synaptic region, requiring only small amounts of the systemic chemoconvulsant to reach the brain [24], [70]. Thus, kainate causes massive CA3 pyramidal cell excitation, which is transmitted to CA1 pyramidal cells via the Schaffer collaterals. Notably, strong excitation mediated via this pathway is in accordance with a modulation of b-AP dynamics in more proximal regions of the CA1 pyramidal cell dendrite (0–200 µm from the soma), as observed in the present study, but not in a rat pilocarpine model which involves exagerated excitatory input to more distal dendritic portions (>200 µm from the soma) directly from the entorhinal cortex [28], [65].

There are two major technical problems with animal models based on systemic chemoconvulsant-induced SE to study intrinsic cellular plasticity. First, these experiments are always biased since only animals which survive SE are used for further experimentation, leading to a possible underestimation of the real brain damage or cellular dysfunction. Moreover, in a slice preparation from an SE-experienced animal the experimenter may pick cells without any alterations and may unwittingly neglect cells with strong alterations in AP dynamics. This creates quite some scattering of the acquired data values (see Fig. S3). Nevertheless, the statistical analysis of our data clearly showed that on average there was an increased likelihood for a reduced somatodendritic excitability immediately after SE. In any case, based on the aforementioned technical limitations, the consequences of SE induction will be underestimated rather than overestimated, meaning that significant differences found in the experimental results are highly likely to reflect real modifications (i.e. intrinsic plasticity). The results of the present study suggest a reduced excitability and spread of dendritic excitation after acute SE. It is unclear whether and, if so, how our results may be related to the occurrence of spontaneous seizures reported for later stages of the model used. Some of the observations made in the present study may be in fact unrelated to epileptogenesis, which implies parallel mechanisms, including, for instance, an initial protection or repair program, maybe of the juvenile brain. A comparison of our experimental results with the ones to be obtained with the same epilepsy model but in the subacute and chronic disease state and/or with adult animals may help to judge which of our findings reflect commencing epileptogenesis and which reflect an acute protection or repair program.

Clinical pharmacology efforts shift from developing anticonvulsants to developing antiepileptic drugs (AEDs) with potential antiepileptogenic or disease-modifying effects. In this context, drugs with potentially prophylactic, neuroprotective, anti-inflammatory, immunosuppressive or neuromodulatory effects have been cosidered [71]. Alternatively, one may pharmacologically suppress or enhance, respectively, intrinsic plasticity processes depending on whether they reflect commencing epileptogenesis and are related to the occurrence of spontaneous seizures or whether they represent a protection or repair program (see above). Thus, blocking Cav3.2 channels at an early stage in order to prevent the development of burst firing and Ca^2+^ overload in neurons has been envisaged as one option. However, the T-type Ca^2+^ channel blocker ethosuximide turned out to be inactive or not preventing epileptogenesis in animal models [71]. Assuming that indications of a strengthening of b-AP attenuation reflect a program that protects CA1 pyramidal cell dendrites from Ca^2+^ overload, any pharmacological intervention which further strengthens b-AP attenuation would be desirable. As our b-AP imaging data suggest, this may be accomplished with the Kv4 current activator NS5806 in CA1 pyramidal cells of the juvenile murine hippocampus. The effects of NS5806 on excitability, AP properties and b-AP-induced Ca^2+^ signal dynamics were in part similar to the ones seen after acute SE (see Fig. S3 and S4). On the other hand, the effects of NS5806 on AP frequency, AP amplitude and ADP integral were opposite to the ones seen after acute SE, and, also unlike acute SE, NS5806 left the AP threshold unaffected. Obviously, during acute SE certain remodeling processes may take place which are not mimicked by NS5806. On the other hand, the action of NS5806 is known to be not restricted to Kv4 channels [39], [72]. Finally, it should also be noted that Kv4 channels do not only mediate *I*_SA_ (Kv4.2 in CA1 pyramidals) but also a transient outward current (*I*_to_) in cardiac myocytes (mostly Kv4.3 in humans; [73]) and that, at least in the dog heart, the NS5806-mediated potentiation of *I*_to_ has been found to recapitulate certain features of Brugada syndrome, a form of cardiac arrhythmia [39]. Thus, NS5806, which we used in an attempt to simulate the results of the present study, is certainly not a good AED candidate. However, compounds related to NS5806 but with a more specific action on Kv4.2 rather than Kv4.3, to be discovered or developed in the future, may become useful AEDs.

In summary, our data provide evidence for intrinsic plasticity mechanisms in CA1 pyramidal cells already active during acute SE. In this time interval detrimental remodeling processes, which reflect the onset of epileptogenesis, may coexist with remodeling processes belonging to a protection or repair program of the brain. Signs of acute alterations of AP dynamics may be related to either of the two scenarios, and appropriate assignment must be made in order to decide whether to suppress or maybe even enhance the observed plasiticity processes. Strengthening of b-AP attenuation may be related to a program that protects dendritic structures from excitotoxicity. Thus, supporting the underlying molecular mechanisms may represent a valuable strategy for antiepileptogenic treatment. Identification of the underlying mechanisms and testing ways of supporting them represent the challenges of future experimental effort.

## Supporting Information

Figure S1 ***I*** **_h_-related subthreshold membrane potential dynamics.** A. Sag time constants as a measure of *I*_h_ activation kinetics (single exponential fits to the voltage relaxation following the negative peak) at different amounts of negative current injection for *sham* (black, 93±3 ms at −200 pA, n = 61) and SE (red, 94±3 ms at −200 pA, n = 71). B. Amplitudes of the voltage sag as a measure of *I*_h_ magnitude (negative peak potential – steady-state potential) at different amounts of negative current injection for *sham* (black, 11.8±0.5 mV at −200 pA, n = 61) and SE (red, 11.7±0.4 mV at −200 pA, n = 71). Data points in A and B are connected by linear regression lines. C. Amplitudes of the voltage sag (*sham*: 4.4 to 19.8 mV, n = 35; SE: 3.7 to 20.6 mV, n = 62) plotted against the negative peak potential (sham: −100 to −147 mV, n = 35; SE: −98 to −142 mV, n = 62; 10 mV binning) obtained at −200 pA current injection as a measure of the voltage dependence of *I*_h_.
(TIF)

Figure S2 **Voltage trajectory between rest and threshold.** For each cell the voltage needed to bring the membrane from rest to threshold was analyzed and mean values calculated for *sham* (17.1±0.9 mV, n = 37), SE (18.6±0.8 mV, n = 61), SE[n] (17.6±1.1 mV, n = 34) and SE[s] cells (20.1±1.4 mV, n = 27; p between all groups >0.05, ANOVA; SE[n] and SE[s], respectively, represent subgroups of data from SE cells that do or do not show an AP notch, see Fig. 4B). The ends of the horizontal bars illustrate mean values for the resting potential (left) and AP threshold (right).
(TIF)

Figure S3 **Statistical analysis of changes in b-AP-induced Ca^2+^ signal dynamics.** A and C. For each individual cell tested with Ca^2+^ imaging the normalized ΔF/F values (see Fig. 6B and 8B) were plotted in logarithmic form against the distance from the soma, and a slope was determined by linear regression; panel A shows the analysis for a *sham* (black) and an SE cell (red), panel C for three different cells, under control conditions (black), in the presence of 20 µM NS5806 (red) and in the presence of 5 mM 4-AP (green), respectively. B and D. Box plots for the slopes (median values) obtained from the different groups, illustrating statistically relevant parameters (upper and lower quartile, sample maximum and sample minimum); panel B: *sham* (black, mean  = −0.0071±0.0004, n = 41) and SE (red, mean  = −0.0093±0.0006, n = 39); panel D: control (black, mean  = −0.0045±0.0003, n = 36), NS5806 (red, mean  = −0.0076±0.0004, n = 15) and 4-AP (green, mean  = −0.0034±0.0003, n = 15; numbers are mean values of the negative slopes in log units/µm); * p<0.05; ** p<0.001 (B: Student's unpaired *t*-test; D: one-way ANOVA and Tukey's multiple comparison test; the difference between SE data in B and NS5806 data in D were also ANOVA-tested and proved to be not significantly different, p>0.05).
(TIF)

Figure S4 **Effects of NS5806 on excitability and AP properties.** Current-clamp experiments were performed on CA1 pyramidal cells (n = 4) from an untreated animal in the absence (Control, black traces and bars) and presence of 20 µM NS5806 (red traces and bars). The holding current between pulses was adjusted to produce a membrane potential of −70 mV A. Voltage deflections elicited by negative current injections (0 to −200 pA for 900 ms). Time points for the measurement of peak potential (circle) and steady-state potential (square) are indicated. B. AP firing elicited by positive current injection (50 pA for 900 ms). C. Membrane time constant (Control: 40.4±2.6 ms, NS5806: 34.4±2.7 ms, p = 0.1062). D. Negative peak potential plotted against current injection and linear fit to calculate R_in_ (Control: 256±25 MΩ, NS5806: 242±36 MΩ, p = 0.7193). E. Steady-state potential plotted against current injection and linear fit to calculate R_in_ (Control: 168±26 MΩ, NS5806: 176±23 MΩ, p = 0.6047). F. AP latency at 50 pA current injection (Control: 132±79 ms, NS5806: 26±6 ms, p = 0.2485). G. AP frequency at 50 pA current injection (Control: 2.8±0.3 Hz, NS5806: 5.0±0.7 Hz, p = 0.0408). H and I. Single somatic APs elicited by a just suprathreshold current injection of 4 ms duration, shown on different time and voltage scales. J. AP threshold (Control: −55.2±1.0 mV, NS5806: −54.9±2.1 mV, p = 0.8412). K. AP amplitude (Control: 118.4±1.8 mV, NS5806: 111.7±1.1 mV, p = 0.0044). L. AP upstroke velocity (Control: 485±30 Vs^−1^, NS5806: 319±28 Vs^−1^, p = 0.0490). M. AP halfwidth (Control: 1.74±0.04 ms, NS5806: 2.38±0.15 ms, p = 0.0153). N. ADP integral (Control: 119±11 mV*ms, NS5806: 59±13 mV*ms, p = 0.0018). O. ADP time constant (Control: 23.0±1.4 ms, NS5806: 13.1±2.9 ms, p = 0.0751). Statistics are based on Student's paired t-tests. Similar to SE, NS5806 left the membrane time constant and R_in_ unaffected, reduced AP upstroke velocity and increased AP halfwidth. Unlike SE, NS5806 left the AP threshold unaffected, increased AP frequency and decreased AP amplitude and ADP integral.
(TIF)
