# Supplementary figures and images for: Acute Alterations of Somatodendritic Action Potential Dynamics in Hippocampal CA1 Pyramidal Cells after Kainate-Induced Status Epilepticus in Mice
Source: PLoS One. 2011 Oct 24;6(10):e26664. doi: 10.1371/journal.pone.0026664 (PMC3200351; doi:10.1371/journal.pone.0026664)

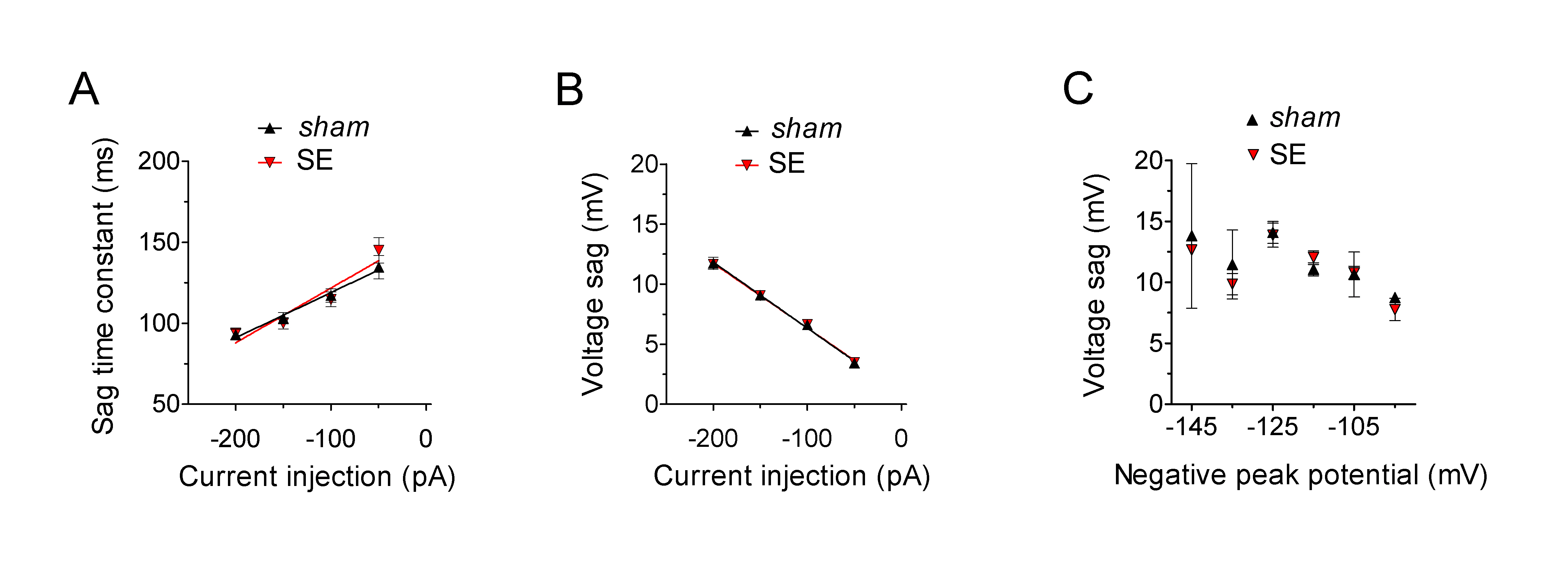

Supplement: Figure S1 — I h-related subthreshold membrane potential dynamics. A. Sag time constants as a measure of I h activation kinetics (single exponential fits to the voltage relaxation following the negative peak) at different amounts of negative current injection for sham (black, 93±3 ms at −200 pA, n = 61) and SE (red, 94±3 ms at −200 pA, n = 71). B. Amplitudes of the voltage sag as a measure of I h magnitude (negative peak potential – steady-state potential) at different amounts of negative current injection for sham (black, 11.8±0.5 mV at −200 pA, n = 61) and SE (red, 11.7±0.4 mV at −200 pA, n = 71). Data points in A and B are connected by linear regression lines. C. Amplitudes of the voltage sag (sham: 4.4 to 19.8 mV, n = 35; SE: 3.7 to 20.6 mV, n = 62) plotted against the negative peak potential (sham: −100 to −147 mV, n = 35; SE: −98 to −142 mV, n = 62; 10 mV binning) obtained at −200 pA current injection as a measure of the voltage dependence of I h. (TIF) [file pone.0026664.s001.tif]

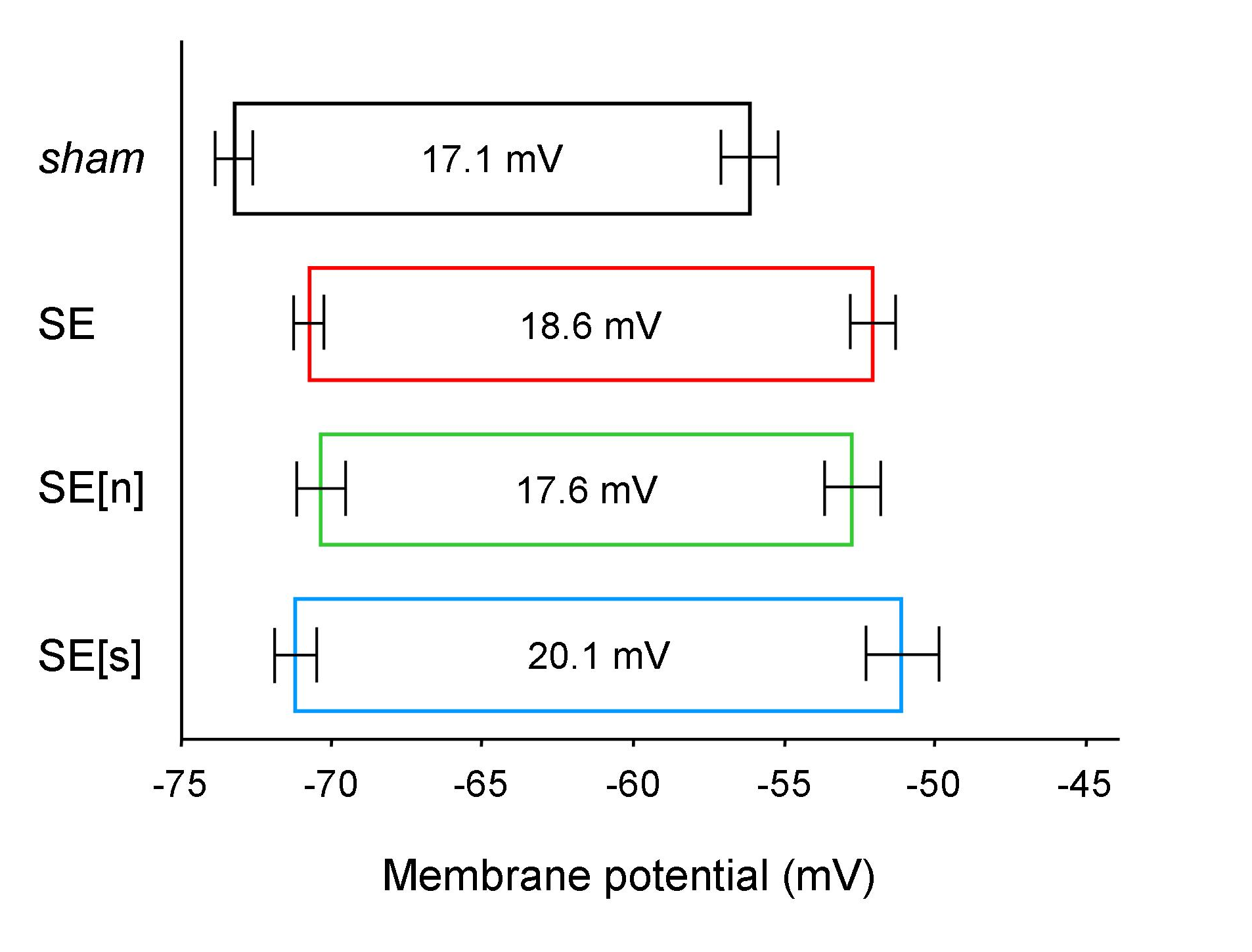

Supplement: Figure S2 — Voltage trajectory between rest and threshold. For each cell the voltage needed to bring the membrane from rest to threshold was analyzed and mean values calculated for sham (17.1±0.9 mV, n = 37), SE (18.6±0.8 mV, n = 61), SE[n] (17.6±1.1 mV, n = 34) and SE[s] cells (20.1±1.4 mV, n = 27; p between all groups >0.05, ANOVA; SE[n] and SE[s], respectively, represent subgroups of data from SE cells that do or do not show an AP notch, see Fig. 4B). The ends of the horizontal bars illustrate mean values for the resting potential (left) and AP threshold (right). (TIF) [file pone.0026664.s002.tif]

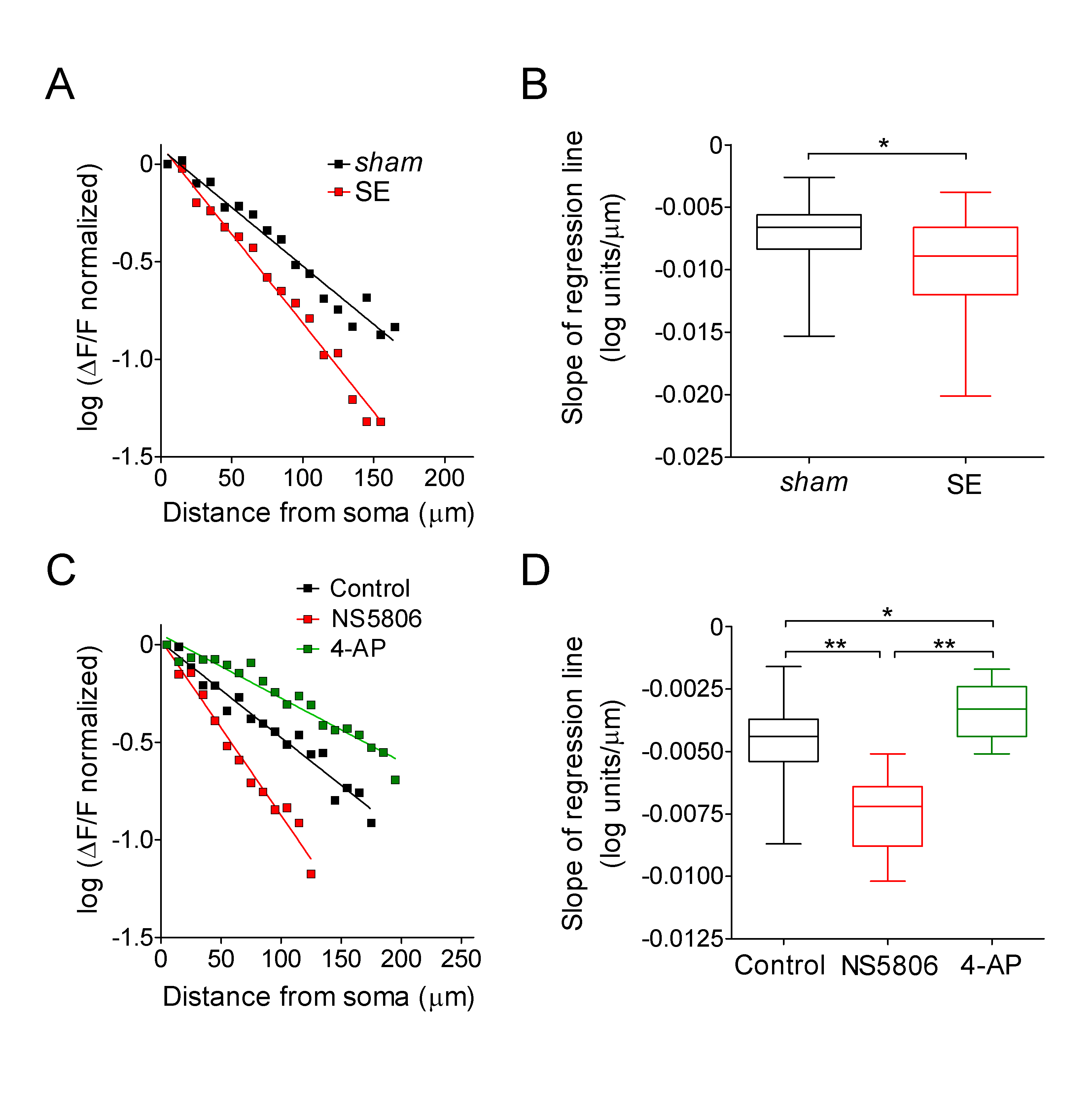

Supplement: Figure S3 — Statistical analysis of changes in b-AP-induced Ca2+ signal dynamics. A and C. For each individual cell tested with Ca2+ imaging the normalized ΔF/F values (see Fig. 6B and 8B) were plotted in logarithmic form against the distance from the soma, and a slope was determined by linear regression; panel A shows the analysis for a sham (black) and an SE cell (red), panel C for three different cells, under control conditions (black), in the presence of 20 µM NS5806 (red) and in the presence of 5 mM 4-AP (green), respectively. B and D. Box plots for the slopes (median values) obtained from the different groups, illustrating statistically relevant parameters (upper and lower quartile, sample maximum and sample minimum); panel B: sham (black, mean = −0.0071±0.0004, n = 41) and SE (red, mean = −0.0093±0.0006, n = 39); panel D: control (black, mean = −0.0045±0.0003, n = 36), NS5806 (red, mean = −0.0076±0.0004, n = 15) and 4-AP (green, mean = −0.0034±0.0003, n = 15; numbers are mean values of the negative slopes in log units/µm); * p<0.05; ** p<0.001 (B: Student's unpaired t-test; D: one-way ANOVA and Tukey's multiple comparison test; the difference between SE data in B and NS5806 data in D were also ANOVA-tested and proved to be not significantly different, p>0.05). (TIF) [file pone.0026664.s003.tif]

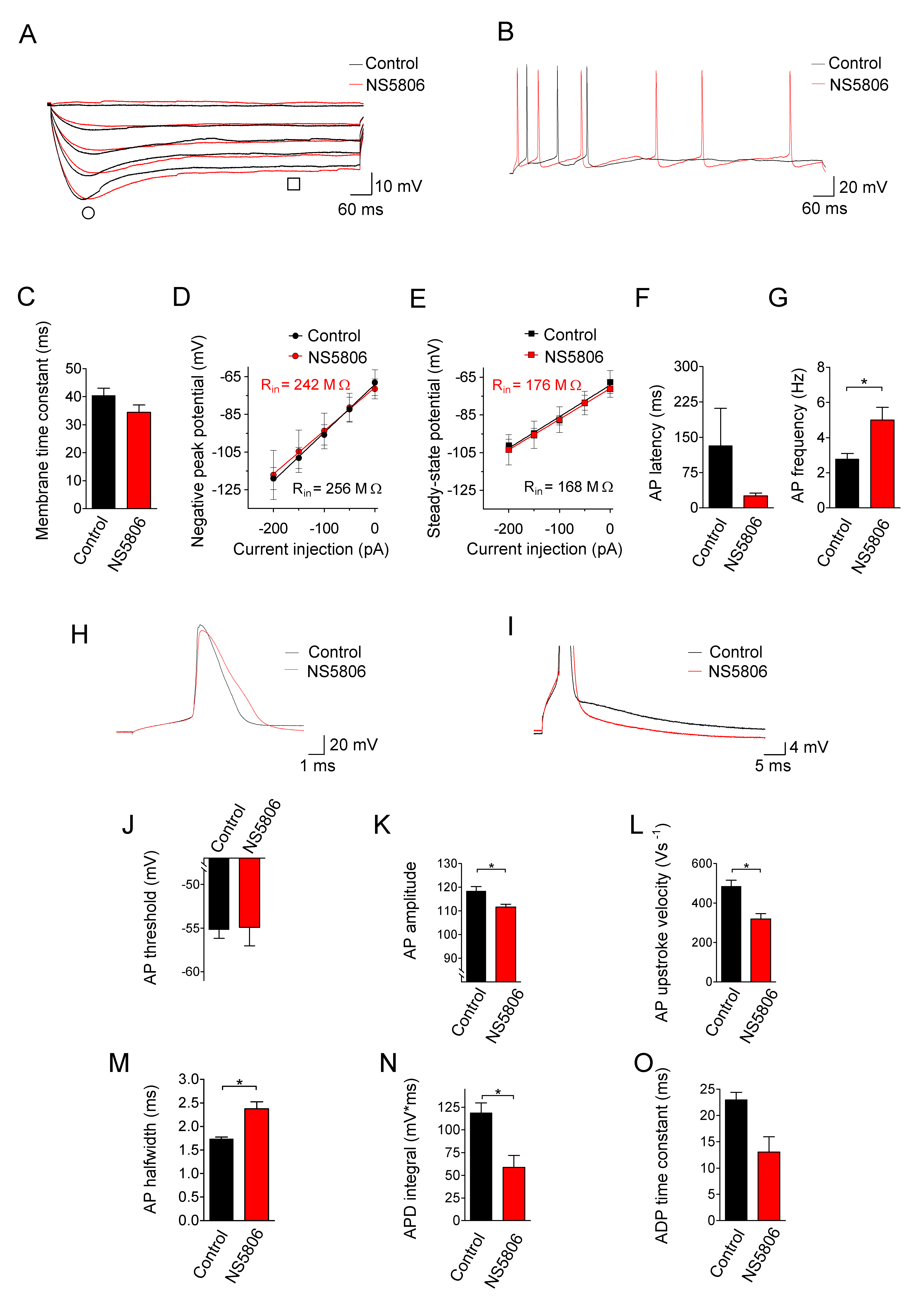

Supplement: Figure S4 — Effects of NS5806 on excitability and AP properties. Current-clamp experiments were performed on CA1 pyramidal cells (n = 4) from an untreated animal in the absence (Control, black traces and bars) and presence of 20 µM NS5806 (red traces and bars). The holding current between pulses was adjusted to produce a membrane potential of −70 mV A. Voltage deflections elicited by negative current injections (0 to −200 pA for 900 ms). Time points for the measurement of peak potential (circle) and steady-state potential (square) are indicated. B. AP firing elicited by positive current injection (50 pA for 900 ms). C. Membrane time constant (Control: 40.4±2.6 ms, NS5806: 34.4±2.7 ms, p = 0.1062). D. Negative peak potential plotted against current injection and linear fit to calculate Rin (Control: 256±25 MΩ, NS5806: 242±36 MΩ, p = 0.7193). E. Steady-state potential plotted against current injection and linear fit to calculate Rin (Control: 168±26 MΩ, NS5806: 176±23 MΩ, p = 0.6047). F. AP latency at 50 pA current injection (Control: 132±79 ms, NS5806: 26±6 ms, p = 0.2485). G. AP frequency at 50 pA current injection (Control: 2.8±0.3 Hz, NS5806: 5.0±0.7 Hz, p = 0.0408). H and I. Single somatic APs elicited by a just suprathreshold current injection of 4 ms duration, shown on different time and voltage scales. J. AP threshold (Control: −55.2±1.0 mV, NS5806: −54.9±2.1 mV, p = 0.8412). K. AP amplitude (Control: 118.4±1.8 mV, NS5806: 111.7±1.1 mV, p = 0.0044). L. AP upstroke velocity (Control: 485±30 Vs−1, NS5806: 319±28 Vs−1, p = 0.0490). M. AP halfwidth (Control: 1.74±0.04 ms, NS5806: 2.38±0.15 ms, p = 0.0153). N. ADP integral (Control: 119±11 mV*ms, NS5806: 59±13 mV*ms, p = 0.0018). O. ADP time constant (Control: 23.0±1.4 ms, NS5806: 13.1±2.9 ms, p = 0.0751). Statistics are based on Student's paired t-tests. Similar to SE, NS5806 left the membrane time constant and Rin unaffected, reduced AP upstroke velocity and increased AP halfwidth. Unlike SE, NS5806 left the AP threshold una [file pone.0026664.s004.tif]
